# Attitudes of Malaysian general hospital staff towards patients with mental illness and diabetes

**DOI:** 10.1186/1471-2458-11-317

**Published:** 2011-05-14

**Authors:** Harry Minas, Ruzanna Zamzam, Marhani Midin, Alex Cohen

**Affiliations:** 1 Centre for International Mental Health, Melbourne School of Population Health, University of Melbourne, Parkville, Victoria 3010, Australia; 2 Department of Psychiatry, Universiti Kebangsaan Malaysia (National University of Malaysia) Medical Centre, Jalan Yaacob Latiff, Cheras Kuala Lumpur, Malaysia; 3 London School of Hygiene and Tropical Medicine, Keppel Street, London WC1E7HT, UK

## Abstract

**Background:**

The context of the study is the increased assessment and treatment of persons with mental illness in general hospital settings by general health staff, as the move away from mental hospitals gathers pace in low and middle income countries. The purpose of the study was to examine whether general attitudes of hospital staff towards persons with mental illness, and extent of mental health training and clinical experience, are associated with different attitudes and behaviours towards a patient with mental illness than towards a patients with a general health problem - diabetes.

**Methods:**

General hospital health professionals in Malaysia were randomly allocated one of two vignettes, one describing a patient with mental illness and the other a patient with diabetes, and invited to complete a questionnaire examining attitudes and health care practices in relation to the case. The questionnaires completed by respondents included questions on demographics, training in mental health, exposure in clinical practice to people with mental illness, attitudes and expected health care behaviour towards the patient in the vignette, and a general questionnaire exploring negative attitudes towards people with mental illness. Questionnaires with complete responses were received from 654 study participants.

**Results:**

Stigmatising attitudes towards persons with mental illness were common. Those responding to the mental illness vignette (N = 356) gave significantly lower ratings on care and support and higher ratings on avoidance and negative stereotype expectations compared with those responding the diabetes vignette (N = 298).

**Conclusions:**

Results support the view that, in the Malaysian setting, patients with mental illness may receive differential care from general hospital staff and that general stigmatising attitudes among professionals may influence their care practices. More direct measurement of clinician behaviours than able to be implemented through survey method is required to support these conclusions.

## Background

Stigmatising attitudes and discriminatory behaviours towards persons with mental illness are of international concern [1,2] and have negative personal and social impact on patients [3-5] and their families [6,7]. Public attitudes towards persons with mental illness often include beliefs that they are dangerous and less capable than the general population [8-10]. Public campaigns to change such views have made inconsistent impact [11,12]. There is a growing literature on negative attitudes and behaviours among health professional and trainees who provide health care. Recognition that such negative attitudes and behaviours among health trainees, and the suggestion by several studies that contact with persons with mental illness may reduce stigma [13-15], has given rise to a series of studies on the effects of mental health training on attitudes [16-20]. However the impact of training in reducing stigma has been inconsistent [18,21,22] and there is uncertainty about the stability of any benefits over time [23]. Some work suggests that those working in mental health settings may have less negative attitudes towards persons with mental illness than do general health care providers [24] while other studies have produced contradictory findings, including findings that mental health workers' attitudes towards persons with mental illness may be no less negative than those of the general public [25].

Studies examining trainee and professional attitudes towards persons with mental illness have used general measures of stigma, primarily measures of social distancing, discrimination and devaluation [19,24,26]. There is a common assumption of a clear link between negative attitudes and discriminatory professional behaviours towards persons with mental illness [e.g., [27,28]]. This assumption has rarely been examined [cf, [29]] and there is reason to question the assumed link between negative attitudes and negative professional behaviours. First, attitude-behaviour relationships are generally weak, regardless of subject matter and settings [30] and, second, behaviour of professionals towards patients is likely to be influenced by multiple situational factors, including clinical role and setting, adherence to professional ethics, and patient characteristics. On the other hand, discriminatory practices by clinicians towards persons with mental illness are common [31,32]. Qualitative studies indicate that persons with mental illness are often dissatisfied with their clinical care, reporting inadequate attention paid to them by clinicians, lack of opportunity for self-determination and involvement in decisions regarding their treatment, and lack of respect shown towards them as people [33-35]. However, the link between generally held attitudes by staff towards persons with mental illness and staff behaviours towards patients is not clear from such studies. Ellsworth [29] made a systematic attempt to explore this link directly by measuring patient reports of staff behaviour towards them and independently assessed attitudes of staff towards mentally ill patients. Staff who scored high on attitudes of restrictive control were more likely to be assessed by patients as insensitive, unfeeling, critical, domineering, and lacking trust in patients. Those staff who scored high on a measure of 'protective benevolence' as a general attitude towards patients were seen by patients as aloof, placating and avoidant. Ellsworth interpreted apparent staff benevolence as a means of maintaining a comfortable interaction with patients rather than having a genuinely humane attitude towards them.

The context of the study is that more people with mental illness are being assessed and treated in general hospital settings by general health staff as part of a global movement away from mental hospitals towards integration of psychiatric services with general health services. Such a reorganisation of mental health services is proceeding rapidly in Malaysia and information about general hospital staff attitudes and behaviours towards persons with mental illness can inform professional training programs and the development of services for people with mental illness.

The purpose of the study was to examine whether general attitudes of hospital staff towards persons with mental illness, and extent of mental health training and clinical experience, are associated with different attitudes and behaviours towards a patient with mental illness than towards a patients with a general health problem - diabetes.

## Methods

The study setting was a large university general hospital in Kuala Lumpur, Malaysia. The study population was all general health professionals (doctors, nurses and paramedical staff) working in this large general hospital.

The study is a between-group design. From the population (all health professionals working at HUKM) two samples were randomly allocated to respond to a questionnaire either about a case of mental illness or a case of diabetes. The questionnaires were identical in all respects except that in the first version of the questionnaire the case vignette to which participants were asked to respond was a case of mental illness and in the second version the case vignette was a case of diabetes. In both vignettes the reason for admission is the same - the patients is "complaining of pressure in the chest". It was expected that random allocation would result in two groups that did not differ significantly on any of the relevant independent variables, including age, sex, ethnicity, professional discipline, total work experience, exposure to mental health and mentally ill patients (amount of mental health training received, amount of work experience in a mental health setting) and level of stigmatising attitudes towards mentally ill persons in general.

At the request of the researchers questionnaires were distributed by heads of clinical departments to all health professional staff in their departments. Heads of departments were given equal numbers of *mental illness *and *diabetes *questionnaires, randomly sorted, for distribution to their staff. Based on information from the human resources department of the hospital about the total numbers of health professional staff, one thousand two hundred questionnaires, plain language statements about the study, and instructions on questionnaire completion and return, were sent to heads of departments for distribution to their staff. It is not known if heads of departments distributed all questionnaires among their staff. Of the 1200 questionnaires distributed 814 (67.8 percent) were returned to researchers. One group of respondents responded to the diabetes vignette (N = 298) and the other to the mental illness vignette (N = 356).

Information about respondents included the respondent's age (years), sex, ethnicity (Malay, Indian, Chinese, Other), health discipline (aggregated into the categories of Medical, Paramedical and Nursing), time in professional employment since first qualified (months), whether and for how long (weeks) the respondent had received psychiatric training, and whether this was part of undergraduate or graduate education, and length of time (weeks) employed in a mental health service setting.

Negative attitudes towards persons with mental illness in general were measured by a questionnaire consisting of seven items that had been derived and adapted from the Opinions about Mental Illness Scale [36] and other relevant 'stigma' questionnaires. The items covered themes of social distancing from persons with mental illness, devaluating persons with mental illness, and belief concerning dangerousness. Responses were on a five-point response scale (*strongly disagree, disagree, not sure, agree, strongly agree*). Preliminary principal components analysis using data from the total sample yielded a single factor underlying the variation in the original set of seven items. Two items were removed to improve the internal consistency of the scale, resulting in an alpha coefficient of 0.65 for the resulting five-item scale.

Attitudes towards a person with mental illness or towards a person with diabetes were measured by asking respondents to read a brief vignette consisting of a brief description of the patient's presenting complaint and their condition over the previous six months [37,38]. For each vignette the 'diagnosis' ('*mental illness' *or '*diabetes mellitus'*) was clearly provided in the instruction set and vignette. (Appendix) The reason given for admission to hospital was the same for the diabetes and mental illness cases: "He is admitted to your ward today complaining of pressure in the chest." Other information included in the two vignettes was made as similar as possible, including a history of self-isolation, argumentativeness/bad temper and regularity of treatment for the background condition. For each vignette respondents were asked to complete a 20-item questionnaire, consisting of a series of clinical behaviour and practice-based attitude statements, that had been developed in a previous study of culture and stigma towards persons with mental illness [39]. Responses were on a five-point scale (*strongly agree, agree, not sure, disagree, strongly disagree*).

Scale construction used all available data. For the main analysis, initial inspection of the data indicated a small group (n = 6) were specialists in mental health and they were removed from further analysis. In the remaining, for 19.0 percent (n = 153) there was missing data on one or more of the variables; this was unsystematic but reduced the analysis sample to 654. The potential biasing of the sample was therefore assessed by comparing the 153 respondents from the left-out group where responses were not missing with the 654 included sample across all variables (background, exposure types, general stigma, response distributions within the general stigma measure, attitudes and which patient type was rated). Using t-tests or chi-square statistics no significant differences were found for any of the variables between the left-out and included groups at the .05 level. Additionally, univariate comparisons conducted for all those responding to a particular variate led to the same result. We conclude that the sample remaining for the main analysis is not a biased subgroup on these variables.

Principal components analysis in the present sample was conducted (with oblique axes rotation to help interpretation) to reduce redundancies and to develop subscales. Five factors were initially identified (eigenvalues > 1.0) of which the first three most directly related to attitudes and care practices, the fourth captured expected patient stereotyped behaviour (e.g., aggression, demanding) and the fifth was defined by a single item. A scree test suggested three or four factors could be retained and so a four-factor solution was refined, leaving out three items that had low communality. The final four-factor solution accounted for 50.51 percent of the variance in item scores and each factor (and no other) had an eigenvalue greater than one. Simple structure was evident with only two items having a non-dominant cross-loading as high as .36 on other factors.

The first factor was labelled Care and Support (items included: "*I would take care, more than usual, to ask Mr. X about his state of health*."; "*More than usual I would ask Mr. X. if he would like to discuss any problems or concerns he is having about his stay in the hospital*."). The resulting final six-item scale had an alpha coefficient of .79. The second factor was labelled Avoidance (items included: "*I would be a little reluctant to work together with Mr. X to develop the care plan*"; "*Compared with other patients, I would avoid confronting Mr. X if he did something against the rules of the ward*."). The resulting five-item scale had an alpha coefficient of .66. The third factor was labelled 'Mistrust' and although originally composed of three items it was reduced to two to improve reliability (alpha = .65). The items were: "*I would not trust the opinion of Mr. X in making treatment decisions*." and "*I would have some doubt that Mr. X could contribute significantly to his management plan*." - which refer to mistrust in the patient's judgements about treatment and care. The fourth factor was composed of three items reflecting expectations of patient behaviour in the ward and included expectations that the patient will be aggressive and demanding, and that the patient's complaint (i.e., felt pressure in the chest) is likely to be 'psychogenic' (implying less legitimacy). The factor was labelled Negative Stereotypes. As a scale, its reliability was low to moderate (alpha = .48) given its low number of items. Overall, five attitude items were removed from further formal analysis, and in any case, additional analysis revealed that these were non-discriminating of the two groups.

Comparison of the two groups (those responding to the diabetes vignette, n = 298, and those to the mental illness vignette, n = 356) was by use of multivariate analysis of variance with follow-up univariate tests. This was supplemented by multivariate analysis of covariance and related univariate tests, taking into consideration any possible differences between groups due to background, exposure and general stigma variables, as well as exploring the influence of such variables on the main outcome variables. Multiple linear regression analyses within each group were conducted to explore the relative influences of background, exposure and general stigma variables on attitude ratings.

Ethics approval was granted by the National University of Malaysia Research Ethics Committee.

## Results

### Sample Characteristics

Table 1 shows comparisons between the two groups on demographic variables, professional discipline, mental health training and experience of working in mental health settings, and general stigma scores. Chi-square analyses revealed no differences in categorical variables (sex, ethnicity, discipline background between groups). For the remaining variables, multivariate analysis of variance indicated no difference between groups overall (Wilks' Lambda = .99, F(5, 648) = 1.37, *ns*) and univariate analyses indicated only slight (non-significant) trends for the diabetes group to have had received more mental health training (F(1, 652) = 3.04, p = .08) and to have worked longer within a mental health setting (F(1, 652) = 3.65, p = .06) than the mental illness group. Thus we conclude that the random allocation of vignettes resulted in reasonably equivalent groups with respect to their background composition and general stigma attitudes.

**Table 1:** Background and exposure characteristics for the two groups

	Diabetes Group (N = 298)	Mental Illness Group (N = 356)	Statistical outcome
**Females **%	286 (96.0)	341 (95.8)	c(1)^2 ^= .01^ns^
**Age**, mean (sd)	28.63 (5.35)	28.04 (5.24)	F(1, 652) = 1.99 ^ns^

**Ethnic Group **%			c(3)^2 ^= 1.37^ns^
Malay	272 (91.2)	317(89.2)	
Chinese	9 (3.0)	13 (3.7)	
Indian	11 (3.7)	19 (5.4)	
Other	6 (2.0)	6 (1.7)	

**Discipline **%			c(2)^2 ^= 5.78^ns^
Medical	22 (7.4)	18 (5.1)	
Paramedical	8 (2.7)	22 (6.2)	
Nursing	268 (89.9)	316 (88.8)	

**Work experience**, months, mean (sd)	67.02 (53.11)	62.29 (54.86)	F(1, 652) = 1.29 ^ns^
**Mental health training**, weeks, mean (sd)	4.06 (13.50)	2.73 (4.77)	F(1, 652) = 3.04 ^ns^
**Mental health work**, weeks, mean (sd)	5.18 (22.94)	2.62 (9.78)	F(1, 652) = 3.65 ^ns^

**General Stigma Score**, mean (sd)	3.28 (.67)	3.29 (.69)	F(1, 652) = .02 ^ns^

ns = not significant (these results were confirmed with t-tests under unequal variances assumptions)

The majority of the sample was female, Malay ethnic background and nurses. The sample was generally young (mean = 28.31, sd = 5.29) and had more than five years of work experience as a health professional (mean = 64.63 months, sd = 54.29). Twenty two percent of the sample had worked in a mental health setting and the mean mental health work experience within this group was 17.2 weeks (sd = 33.16). The respondents had received a mean of 3.79 (sd = 9.78) weeks of training in mental health, with 54.7 percent having received some training. The majority of those who had received mental health training (97.5%) indicated that their training was at an undergraduate level. Mean scores on the general stigma measure indicated a tendency towards agreement (mean = 3.29, sd = .68), although there was substantial variation in levels of agreement on the items composing the general stigma scale.

### General stigma towards persons with mental illness

Preliminary analyses comparing the two groups (mental illness group and diabetes group) on their item responses indicated no substantial difference between the groups in level of agreement with the items of the general stigma scale. Thus in Table 2 we summarise results for the two groups combined Level of agreement across the five items ranged from a third to more than 70 percent of the sample. Cross-item variation was tested by mixed analysis of variance with repeated measures on items and including group as a between-subjects factor. Given that the sphericity assumption was not met the multivariate outcome is reported which indicated a significant variation in responses across items (Wilks' Lambda = .219, F(4,627) = 560.41, p < .0005). Groups did not differ in ratings nor was the item by group interaction significant. Pair-wise comparisons using repeated measures t-tests among items revealed significant differences among all items (p < .05) and after Bonferroni adjustment (alpha = .005) between all except items 2 and 3 (Table 2).

**Table 2:** Percent agreeing with items measuring general stigma

Item	% Agree	% Strongly Agree	% Overall Agreement
1. A woman would be foolish to marry a man who has had a severe mental illness, even though he seems to be fully recovered.	25.9	7.7	33.6
2. It is frightening to think of people with mental problems living in your own neighbourhood.	32.4	4.0	36.4
3. Someone with a history of mental illness should not be given a job of high responsibility.	41.3	6.0	47.3
4. Although some mental patients seem all right, it is dangerous to forget for a moment that they are mentally ill.	59.4	7.5	67.1
5. A woman who has had a history of mental illness should not be hired as a babysitter.	52.4	19.0	71.4

Mean	42.3	8.8	51.1

### Comparisons of attitudes

Table 3 summarises findings in relation to attitudes, including statistical outcomes in conducting comparisons between raw measures (by analysis of variance) and residualised scores (by analysis of covariance). First, multivariate analysis of variance indicated a significant difference between groups (Wilks' Lambda = .95, F(4, 649) = 8.83, p < .005). Univariate follow-up tests as indicated in Table 3 show that Care and Support scores were significantly lower in ratings of the mental illness case relative to the diabetes case and significantly higher Avoidance and Negative Stereotypes scores for the mental illness case ratings. No differences were evident in Distrust scores. Analysis was repeated using as covariates background factors (age, sex, total work experience), exposure factors (amount of mental health training and amount of work experience in a mental health setting) and general stigma scores. Results were the same as before in indicating lower Care and Support and higher Avoidance and Negative Stereotypes endorsed for the mental illness than the diabetes case. Several covariate effects were significant (Table 3, notes) including for Care and Support the factors of general stigma, professional discipline and gender. Care and Support scores increased with higher general stigma scores, being a nurse rather than another professional discipline, and for females. For Avoidance scores significant covariate effects included general stigma score and professional discipline (in this case *not *being a nurse was related to higher Avoidance scores). Mistrust scores, though not different between groups, were also influenced by covariates, but primarily general stigma (the effect of amount of mental health work very small). Negative Stereotype expectations were endorsed more by those with higher scores on general stigma, and by nurses more than by other professional disciplines. In general, it does appear that general stigma is related to attitudes towards the specific cases but when this is held constant across groups those rating the case of mental illness endorsed lower Care and Support and higher Avoidance and Negative Stereotype expectations of the patient than those rating the diabetes case.

**Table 3:** Group comparisons on attitude measures

Scale	Diabetes Group (N = 298)	Mental Illness Group (N = 356)	ANOVA F	ANCOVA F
**Care & Support**	3.56 (.68)	3.41 (.71)	7.86 (p < .01)	6.63 (p < .05)
**Avoidance**	2.50 (.59)	2.64 (.57)	8.59 (p < .01)	7.58 (p < .01)
**Mistrust**	2.88 (.85)	2.98 (.82)	2.03 ^ns^	2.60 ^ns^
**Negative Stereotypes**	3.23 (.66)	3.39 (.60)	10.04 (p < .01)	10.29 (p < .01)

ns = not significant;

Significant covariate effects and direction of effects:

*Care & Support*: higher general stigma (F(1, 645) = 15.08, p < .0005); is a nurse (F(1, 645) = 68.89, p < .0005); is female (F(1, 645) = 4.70, p < .05);

*Avoidance*: higher general stigma (F(1, 645) = 35.09, p < .0005); is a not a nurse (F(1, 645) = 22.59, p < .0005);

*Mistrust*: higher general stigma (F(1, 645) = 32.58, p < .0005); more MH work experience (F(1, 645) = 4.01, p = .046)

*Negative Stereotypes*: higher general stigma (F(1, 645) = 36.47, p < .0005); is a nurse (F(1, 645) = 15.15, p < .0005).

It should be kept in mind that the overall ratings for the three attitudes fall in the region of agreement in relation to Care and Support (mean = 3.48, sd = .70) and disagreement with Avoidance (mean = 2.58, sd = .58) and Mistrust (mean = 2.93, sd = .84), as might be expected from a sample of health professionals. Unexpectedly, Negative Stereotypes tended to be agreed with for both patient types, perhaps reflecting the specific vignette content highlighting aggressiveness/bad temper in these patients (mean = 3.32, sd = .63).

### Factors related to attitudes

Multiple regression analyses were conducted to explore the unique predictors of attitudes within each of the groups rating the two vignettes. The intent was to explore if general stigma is associated with attitudes towards the specific cases when controlling for other factors. Although the covariance analysis suggests this is the case, the regression analyses serve to indicate if this relationship is restricted, as expected, to the mental illness case ratings. Table 4 shows the zero order correlation between attitudes and predictor variables along with beta coefficients and their associated t-values. First, it is clear from inspection of Table 4 that general stigma is associated with attitudes to specific cases, while holding other factors constant. For the mental illness group (upper panel) higher general stigma score is associated with higher scores on all attitude scales, including Care and Support. More surprising is that the findings are the same for the diabetes case.

**Table 4:** Results of regression analyses examining background and general stigma as predictors of attitudes

	Mental Illness Vignette Group											
	**Care & Support**			**Avoidance**			**Mistrust**			**Negative Stereotypes**		

**Predictors**	**r**	**Beta**	**t-value**	**r**	**Beta**	**t-value**	**r**	**Beta**	**t-value**	**r**	**Beta**	**t-value**

Gen stigma	.19***	.10	2.02*	.13*	.19	3.60***	.25***	.23	4.37***	.25***	.19	3.77***
Sex	.10	-.12	2.09*	-.03	.11	1.76	.05	-.04	< 1	.07	-.10	1.61
MH work	-.06	-.01	< 1	.00	-.01	< 1	-.04	.00	< 1	-.08	-.04	< 1
MH training	-.03	.02	< 1	-.05	-.07	1.42	-.09	-.08	1.52	.03	.06	1.24
Age	.02	.22	1.82	.03	-.10	< 1	.09	.07	< 1	-.09	.09	< 1
Work experience	.01	-.11	< 1	.03	.07	< 1	.09	.04	< 1	-.09	-.12	1.00
Discipline	.38***	.45	7.55***	-.22***	-.33	5.35***	.12*	.10	1.63	.28***	.29	4.73***

**Model F**	10.78***			5.30***			4.64***			7.55***		
**Adj R^2^**	.162			.078			.067			.114		

	**Diabetes Vignette Group**											

Gen stigma	.24***	.20	3.56***	.28***	.29	4.98***	.21***	.22	3.69***	.28***	.27	4.64***
Sex	.10	0.05	< 1	.01	.03	< 1	.01	.03	< 1	.07	-.03	< 1
MH work	.03	.04	< 1	-.04	-.03	< 1	.10	.12	2.09*	-.04	.00	< 1
MH training	.05	.09	1.60	-.05	-.02	< 1	-.07	-.08	1.31	-.10	-.04	< 1
Age	-.05	-.07	< 1	.03	.09	< 1	.06	.03	< 1	-.14*	-.30	2.59*
Work experience	-.01	.09	< 1	.00	-.09	< 1	.07	.04	< 1	-.06	.22	1.97*
Discipline	.26***	.27	3.79***	-.02	-.09	1.23	-.01	-.05	< 1	.14*	.06	< 1

**Model F**	5.71***			3.92***			3.03**			5.20***		
**Adj R^2^**	.100			.064			.046			.090		

*p < .05; **p < .01; **p < .001; r = zero order correlation

A regression analysis was conducted exploring the predictors (as listed in Table 4) of general stigma scores from among the other factors in the overall sample. The model was significant although it explained a low proportion of the variance in general stigma scores (F(7, 647) = 6.06, p < .001, Adjusted R^2 ^= .044). The only predictor of general stigma was professional discipline (Beta = .22, t = 4.68, p < .0005), showing higher scores among nurses than the other professional groups.

## Discussion

Use of a between-group design, with random allocation of the mental illness and diabetes vignettes, was an effective strategy in generating two groups that were essentially equivalent on general stigma scores, demographic variables, and levels of training and experience in mental health. Comparison of the respondents who provided complete data with those who were excluded from analysis on the basis of incomplete data showed that there was no significant difference between the groups on general stigma scores, demographic variables, and levels of training and experience in mental health. There is reason to be confident, therefore, that results from the sample included in the analysis are representative of the total (n = 814) respondents and, given the high response rate to the survey, perhaps to the overall hospital health professional workforce.

Despite the fact that the patients described in the two vignettes were admitted to hospital for the same somatic complaint attitudes towards them by health professionals were significantly different, with lower scores on Care and Support and higher Avoidance and Negative Stereotype expectations in relation to the mental illness case than to the diabetes case.

General stigma, controlling for other variables, was associated with attitude ratings, and was associated with higher scores on all attitude subscales. While Avoidance and Negative Stereotyping may be logically expected to be related to general stigma towards persons with mental illness, as reflected in the social distancing measure, it is perhaps more surprising that Care and Support ratings were positively associated with general stigma. It may be that those with higher endorsement of general stigma had higher expectations of deficit in the mental illness case because the social distancing measure also captures perceptions of incapability in persons with mental illness.

The regression analyses examined whether the relationship between general stigma and attitudes was limited to the mental illness case, with the expectation that there would be no systematic association between these in the diabetes case. However, the relationship held true for the diabetes as well as the mental illness case. We have no explanation for this effect other than the measure of general stigma may tap into a more general factor related to hospital staff attitudes towards people with any illness. An attempt to explore the variance explained in general stigma ratings from the limited demographic and exposure factors proved to be of limited worth with only a small proportion of the variance explained by discipline background of the respondent. Further research is needed to explore why health professionals endorsing higher social distancing of persons with mental illness are also more likely to endorse higher care and support, higher avoidance, higher negative stereotype expectations, and higher mistrust of a patients with a somatic problem (diabetes).

It is clear from the findings that mental health work experience and mental health training were not associated with variations in general stigma or attitude ratings, despite the fact that the 22 percent of the sample that had worked in a mental health setting had done so for a substantial period, a mean of 17 weeks. The lack of effect of training level could be attributed to the fact that such training generally occurred a number of years previously and mostly in undergraduate courses, so that even if there had been an impact of such training and experience [40] impact was not sustained. This finding, that experience of working in a mental health setting was not related to attitudes towards persons with mental illness or to professionals' practice orientations, contradicts the contact hypothesis [19].

It is important to note that a negative relationship was not observed between experience and the dependent variables, as it is possible that exposure to negative experiences with those having mental illness (e.g. episodes of aggression, etc.) may have served to affirm negative attitudes towards persons with mental illness [e.g., [41]] thereby influencing attitudes. In future research it is important to explore directly the qualitative aspects of experience in mental health settings [e.g., [41,42]] in the Malaysian context and how these may be related to attitudes towards persons with mental illness.

Professional discipline was associated with ratings of general stigma as well as ratings of attitudes to the cases in the vignettes. Nurses, compared with medical and paramedical staff, had higher general stigma scores and higher Care and Support, lower Avoidance and higher Negative Stereotype expectations in relation to patients. The explanation for the nurses' higher general stigma score is unclear. Differences may be a function of personal opportunity and predilection in career choice, training, clinical exposures or clinical roles adopted by the different disciplines in the Malaysian setting. Further comparative research is needed, first, to confirm the discipline effect, particularly since the non-nursing sub-sample was very small, and second, to elucidate factors that may have led to different attitudes among the professional disciplines towards persons with mental illness.

Turning to the discipline differences in relation to attitudes, the regression analyses clarified that higher Care and Support ratings by nurses than others were evident for both the mental illness and diabetes vignettes. This may have to do with the higher intensity of contact with patients that is characteristic of the nursing role. Perhaps role compatibility of the items in our measure played a role. Most items highlight an interactive, social care dimension in treatment (e.g., supporting the patient in self-care, supporting families, influencing the family to support the patient, resolving issues about patient comfort during the admission) that may be more compatible with the nursing role than with the roles of medical and paramedical staff. The regression analyses however also indicated that lower Avoidance and higher Negative Stereotype expectations were endorsed by nurses in the mental illness case but not in the diabetes case. This was unrelated to general stigma as this was controlled statistically. Lower Avoidance within the nursing group may be because of their higher Care and Support towards the case with mental illness. However, through post-hoc inquiry within the nursing group, these two scales were positively correlated (r (316) = .19, p < .01) Nurses who endorsed higher Care and Support also endorsed more Avoidance. This was different for medical and paramedical group where higher Care and Support was negatively correlated with Avoidance (r (40) = -.41, p < .01) regarding the mental illness vignette. It would appear that nurses express a more ambivalent role towards the mental illness vignette, combining apparently conflicting orientations of need to provide more intensive care than usual and greater reluctance to engage with the patient [43] compared with other health professionals. The latter appear to position themselves more clearly along a Care and Support versus Avoidance dimension. It remains unclear why nurses endorsed less Avoidance and higher Negative Stereotype expectations. This may be due to the requirements of their clinical role of providing care through their more intensive and continuing contact with the patient than is the case for the other professional groups,.

Finally, This seems to be the only study that has examined general stigma towards persons with mental illness among general hospital health professionals. In a study of attitudes among Malaysian medical students using the Attitudes Towards the Mentally Ill scale [20] low proportions of students agreed with stigmatising attitudes. In an item similar to the one used here, that a mentally ill patient should not be given a job with high responsibility, only 11 percent agreed with this before their psychiatry training and 15 percent after the training (compared with 47% in the current sample). In a study of stigma among nurses in Melbourne [39], using the same general stigma scale as in this study, Anglo-Australian and Chinese background nurses working either in general health or in mental health were compared. In that work 42.8 percent of Chinese nurses on average agreed with the items on the scale compared with 15.1 percent of Anglo-Australian nurses, suggesting possible cultural differences. Less mental health nurses agreed than general nurses (20.8% versus 37.0%) indicating effects of specialization. More pertinent to the present study was that among general nurses 55.5 percent of the Chinese agreed with the general stigma items, comparable to the rate seen in the present sample (51.1%, Table 2) while only 15.1 percent of Anglo-Australians did so. (The comparable figures among mental health nurses were 30.0 and 11.6 percent respectively). There is a need for further study of possible cultural influences on ratings of general stigma among general health professionals [9].

The present study suggests that there is a need to review the mental health training of general health staff and to consider ways in which differential attitudes and behaviours towards persons with mental illness can be reduced.

## Conclusions

General hospital staff report different attitudes, and suggest that they would behave differently, towards patients with diabetes and patients with mental illness, even when they present to hospital with a similar somatic complaint. It should be highlighted that it was not actual behaviours of staff in relation to real patients that were studied but self-reported attitudes and intended actions in response to brief clinical vignettes. While the use of brief written vignettes is common in stigma research the method has significant limitations. Whether general clinical staff behave in a discriminatory fashion [33-35] towards person with mental illness can only be answered by studying the actual behaviour of hospital staff towards real patients.

As expected, a more negative attitude towards persons with mental illness in general (as measured by responses to the general stigma questionnaire, Table 2) was associated with higher ratings on Care and Support, Avoidance and Negative Stereotype in relation to the mental illness case than for the diabetes case. However, we unexpectedly found that negative attitudes towards persons with mental illness in general were also associated with more negative attitudes towards the diabetes case. It is possible that the measure of general stigma may tap into a more broadly applicable dimension of negative attitudes towards people with any illness.

Finally, extent of mental health training (as part of general health training) and duration of experience of working in mental health settings did not influence attitudes or social distancing in relation to mental illness. Training influences could be expected to be minimal because most respondents had received only limited mental health training during their undergraduate education. The lack of effect of experience of working in mental health settings contradicts the common claim that contact with persons with mental illness reduces negative attitudes and stereotypes.

## Appendix 1: Vignettes

### Vignette 1

Please read the following description of Mr. X who suffers from mental illness and answer the questions that follow. Mr. X is 24 years old and lives at home with his parents. He has had a few temporary jobs since leaving school. He is currently unemployed. Over the past six months, he has more or less stopped seeing his friends and spends most of his time in his room at home. He neglects to do chores at home and when his parents request this from him, he loses his temper easily. Even if he is alone in his room, his parents have often heard him arguing as if someone else is there. He has regular follow up in the psychiatric clinic and is taking psychotropic medications. He is admitted to your ward today complaining of pressure in the chest.

### Vignette 2

Please read the following description of Mr. X who suffers from diabetes and answer the questions that follow. Mr. X is 24 years old and lives at home with his parents. He has suffered from diabetes for the last two years and his condition has not been under sufficient control for most of this period. After the onset of his illness, he has held only a few temporary jobs. He is presently unemployed. He has progressively lost his self-confidence and has tended to stay in his room at home and not do much around the house. His parents say that his temper has been increasingly bad over the last three months. Nevertheless, he remains to be in contact with a few friends but has tended not to go out as much as he used to. He attends his outpatient clinic appointment for his diabetes problem regularly and is on medications for the condition. He is admitted to your ward today complaining of pressure in the chest.

## Competing interests

The authors declare that they have no competing interests.

## Authors' contributions

All authors were involved in the conceptualization and design of the study. HM and SM (see acknowledgements) constructed the survey questionnaire. Data collection was coordinated by RZZ and MM. Data analysis and interpretation were carried out by SM and HM. The draft manuscript was written by HM and SM. All authors contributed to the writing of, and approved, the final version of the manuscript.

## Pre-publication history

The pre-publication history for this paper can be accessed here:

http://www.biomedcentral.com/1471-2458/11/317/prepub
